# Tumour-derived exosomal lncRNA-SOX2OT promotes bone metastasis of non-small cell lung cancer by targeting the miRNA-194-5p/RAC1 signalling axis in osteoclasts

**DOI:** 10.1038/s41419-021-03928-w

**Published:** 2021-07-02

**Authors:** Jianjiao Ni, Xiaofei Zhang, Juan Li, Zhiqin Zheng, Junhua Zhang, Weixin Zhao, Liang Liu

**Affiliations:** 1 Department of Radiation Oncology, Fudan University Shanghai Cancer Center, Shanghai, China; 2 Department of Oncology, Shanghai Medical College, Fudan University, Shanghai, China; 3 Department of Radiation Oncology, Fudan University Shanghai Cancer Center Minhang Branch Hospital, Shanghai, China

**Keywords:** Non-small-cell lung cancer, Oncogenesis

## Abstract

Bone is a frequent metastatic site of non-small cell lung cancer (NSCLC), and bone metastasis (BoM) presents significant challenges for patient survival and quality of life. Osteolytic BoM is characterised by aberrant differentiation and malfunction of osteoclasts through modulation of the TGF-β/pTHrP/RANKL signalling pathway, but its upstream regulatory mechanism is unclear. In this study, we found that lncRNA-SOX2OT was highly accumulated in exosomes derived from the peripheral blood of NSCLC patients with BoM and that patients with higher expression of exosomal lncRNA-SOX2OT had significantly shorter overall survival. Additionally, exosomal lncRNA-SOX2OT derived from NSCLC cells promoted cell invasion and migration in vitro, as well as BoM in vivo. Mechanistically, we discovered that NSCLC cell-derived exosomal lncRNA-SOX2OT modulated osteoclast differentiation and stimulated BoM by targeting the miRNA-194-5p/RAC1 signalling axis and TGF-β/pTHrP/RANKL signalling pathway in osteoclasts. In conclusion, exosomal lncRNA-SOX2OT plays a crucial role in promoting BoM and may serve as a promising prognostic biomarker and treatment target in metastatic NSCLC.

## Introduction

Bone metastasis (BoM) is one of the important factors leading to increased rates of disease recurrence and mortality in cancer patients, and it occurs frequently in lung cancer, breast cancer and prostate cancer [1]. The incidence of BoM in non-small cell lung cancer (NSCLC) is reported to be as high as 66.7% [2, 3]. BoM can easily cause pain, hypercalcaemia, spinal cord compression, pathological fractures and nerve compression symptoms [4], which present considerable challenges to patient survival and quality of life.

The vast majority of NSCLC BoM lesions are osteolytic, which manifest as bone tissue dissolution, destruction and resorption. Tumour cells can stimulate the activation of osteoclasts, which are the key mediators of the bone resorption process [5]. Mechanistically, NSCLC cells can stimulate the receptor activator of NF-κB (RANK) pathway in osteoblasts by altering the microenvironment of the bone tissue, allowing the body to misjudge the bone balance. Subsequently, RANK binds to RANK ligand (RANKL) and promotes the maturation of osteoclasts, which can play a devastating role in osteolysis [6]. Hence, overexpression of RANKL can increase bone resorption. On the other hand, the naturally secreted decoy receptor osteoprotegerin (OPG) inhibits osteoclast maturation and activation, inducing osteoclast apoptosis and inhibiting bone resorption [7, 8].

In the process of osteolytic BoM, the bone matrix secretes a large amount of transforming growth factor-β (TGF-β) [9], which promotes the production of parathyroid hormone related protein (pTHrP) and further stimulates RANKL expression [10]. This enhances the activation of osteoclasts and induces an increase in abnormal bone resorption, eventually causing osteolytic BoM. Therefore, TGF-β/pTHrP/RANKL signalling plays a vital role in promoting osteoclast differentiation and BoM in NSCLC. However, the upstream regulatory mechanism of TGF-β/pTHrP/RANKL signalling has not been completely clarified.

In recent years, accumulating evidence has indicated that exosomes are closely related to tumour metastases, and exosomes can reach the site of metastasis before tumour cells do, change the microenvironment, activate the biological function of cells, and thereby promote tumour metastasis [11]. Exosomes carry a variety of molecules, including mRNAs, miRNAs, circRNAs and lncRNAs [12–14]; mediate cell-to-cell communication; affect the extracellular environment; and regulate downstream gene expression. However, studies focusing on the regulation of BoM by exosomes in NSCLC are limited [15].

LncRNA-SOX2OT is a recently characterised tumour-promoting long noncoding RNA that plays critical roles in diverse human cancers, including pancreatic cancer [16, 17], hepatocellular carcinoma [18], glioblastoma [19] and osteosarcoma [20, 21]. In NSCLC, lncRNA-SOX2OT expression was found to be upregulated in tumour tissue and serum samples, therefore, its expression level has valuable prognostic significance [22]. Moreover, lncRNA-SOX2OT was demonstrated to be enriched in exosomes from various cancers including NSCLC [23], and exosomal transfer of lncRNA-SOX2OT plays crucial roles in regulating tumorigenesis [16]. However, the relationship between lncRNA-SOX2OT and BoM and the exact role of exosomal lncRNA-SOX2OT in NSCLC have not been examined.

In this study, we test our hypothesis that NSCLC cell-derived exosomal lncRNA-SOX2OT can modulate osteoclast differentiation and stimulate BoM by regulating the TGF-β/pTHrP/RANKL signalling pathway in osteoclasts. The prognostic significance of exosomal lncRNA-SOX2OT and its regulatory effects on osteoclast differentiation and BoM in NSCLC are investigated, and the potential molecular targets and underlying mechanisms are also explored. Our data will show the clinical significance that exosomal lncRNA-SOX2OT may serve as a powerful prognostic biomarker for NSCLC patients with BoM and uncover a molecular mechanism by which NSCLC cell-derived exosomal lncRNA-SOX2OT modulates osteoclast differentiation and stimulates BoM by targeting the miRNA-194-5p/RAC1 signalling axis and TGF-β/pTHrP/RANKL signalling pathway in osteoclasts.

## Results

### Exosomal lncRNA-SOX2OT expression is upregulated in NSCLC patients with BoM and correlates with shortened survival

**Fig. 1:**
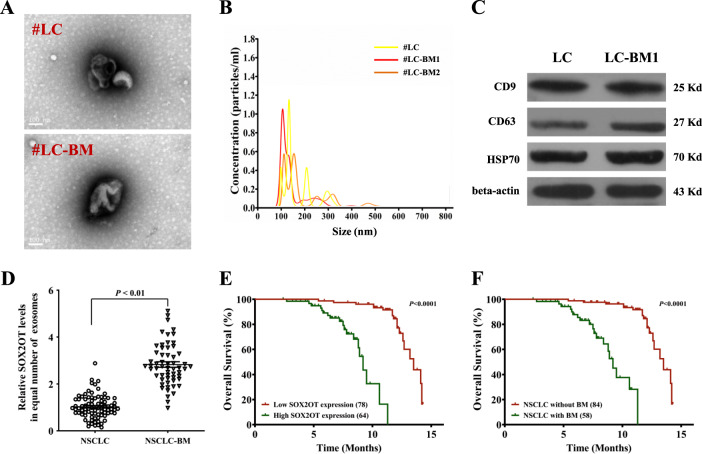
LncRNA-SOX2OT-enriched exosomes in the plasma of NSCLC patients correlate with BoM. **A** Exosomes derived from the plasma of NSCLC patients with or without BoM were phenotyped (purity and shape) by transmission electron microscopy. Scale bar, 100 nm. **B** The size and particle count of the indicated exosomes with an LM10 nanoparticle characterisation system. Data are representative of three biological replicates. The representative pictures are presented. **C** Western blotting was performed to detect the expression of exosomal markers, CD9, CD63 and HSP70. Beta-actin was used as a reference protein. Data are representative of three biological replicates. **D** A real-time PCR assay was performed to determine the lncRNA-SOX2OT level in exosomes derived from the plasma of lung cancer patients with or without BoM. GAPDH was used as the internal control. **E** The Kaplan-Meier analysis was executed to determine the correlations between BoM and OS. **F** The Kaplan–Meier analysis was also performed to determine the correlations between the level of lncRNA-SOX2OT in exosomes and OS. *, *P* < 0.05; **, *P* < 0.01 (*t* test). Data are representative of three biological replicates.

Exosomes were extracted from the plasma of NSCLC patients with or without BoM. Transmission electron microscopy detection demonstrated that the purified exosomes exhibited a typical cup-shaped morphology (Fig. 1A). Next, the LM10 nanoparticle characterisation system was employed to measure the dimensions of the exosomes, and we found that the exosomes were successfully purified with feature sizes ranging from 100 to 200 nm (Fig. 1B). More than 2 × 10^7^ particles/ml exosomes were present in plasma samples from all NSCLC patients (Fig. 1B). Little difference was observed in the exosome quantity between NSCLC patients with and without BoM. Positive expression of exosome markers including CD9, CD63 and HSP70 was confirmed (Fig. 1C, Figure S1). Next, a real-time PCR assay demonstrated that more lncRNA-SOX2OT was accumulated in the exosomes from the peripheral blood of patients with BoM than in those of patients without metastasis (Fig. 1D). We then analysed the survival of NSCLC patients with or without BoM and found that NSCLC patients with BoM had shorter overall survival (Fig. 1E). Moreover, the levels of lncRNA-SOX2OT in NSCLC patient-derived exosomes were inversely correlated with overall survival (Fig. 1F). Overall, lncRNA-SOX2OT expression was upregulated in exosomes from NSCLC patients with BoM, and lncRNA-SOX2OT may serve as a powerful prognostic biomarker.

### LncRNA-SOX2OT-enriched exosomes promote NSCLC BoM

**Fig. 2:**
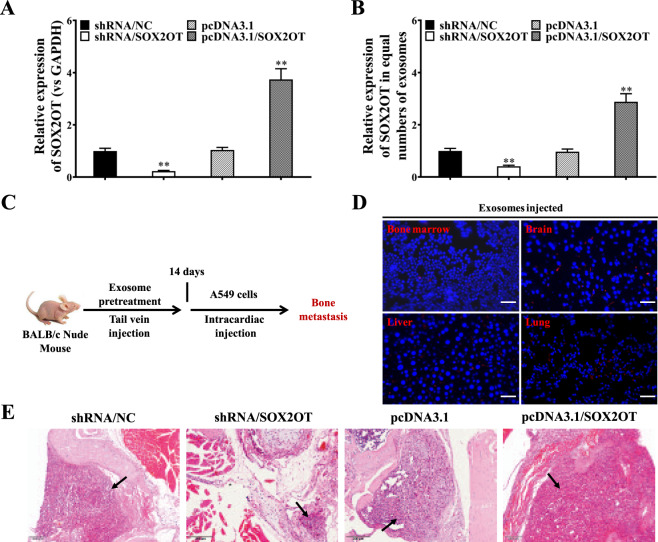
LncRNA-SOX2OT-enriched exosomes promote NSCLC BoM. **A**, **B** LncRNA-SOX2OT expression was evaluated by real-time PCR in the cytoplasm and exosomes of lncRNA-SOX2OT-knockdown and lncRNA-SOX2OT-overexpressing A549 cells and their respective controls. GAPDH was used as the internal control. Data are representative of three biological replicates. **C** The protocol for studying the tissue distribution of exosomes is shown. Exosomes were administered to BALB/c nude mice by tail vein injection. **D** In vivo tracing of injected exosomes in the lungs, liver, bone marrow and brain of mice was performed. RAW264.7 cells were incubated with PKH-26-labelled exosomes (red fluorescent) derived from A549 cells. Nuclei were stained with 4′,6-diamidino-2-phenylindole (DAPI). The incorporation of red fluorescent exosomes into the targeted RAW264.7 cells was observed under a fluorescence microscope. **E** For the NSCLC BoM experiment, BALB/c nude mice were pre-treated with exosomes (10 μg/mouse) containing different amount of lncRNA-SOX2OT every other day for 4 weeks by tail vein injection. Subsequently, A549 cells were injected into the left cardiac ventricle to establish experimental BoM models. The effect of lncRNA-SOX2OT-enriched exosomes on BoM was analysed by staining tissue sections with H&E. Black arrows indicate the metastatic foci and cancerous cells in the representative images. Scale bar, 200 μm. Each experiment was performed at least three times independently. *, *P* < 0.05; **, *P* < 0.01 (t test).

To determine the function of lncRNA-SOX2OT, we manipulated the expression of lncRNA-SOX2OT in NSCLC cells. As shown in Fig. 2A, lncRNA-SOX2OT was successfully knocked down and overexpressed in A549 cells. Moreover, we found that lncRNA-SOX2OT levels were reduced in exosomes from lncRNA-SOX2OT-knockdown A549 cells but elevated in those from lncRNA-SOX2OT-overexpressing A549 cells (Fig. 2B). Next, to study the in vivo distribution of exosomes, purified exosomes were injected into BALB/c nude mice by tail vein injection according to published protocols (Fig. 2C). In vivo tracing of PKH-26-labelled exosomes showed that NSCLC-derived exosomes were distributed in the lungs, liver, bone marrow and brain (Fig. 2D). Next, to reproduce BoM of the NSCLC in an animal model experimentally, BALB/c nude mice were pre-treated with exosomes containing lncRNA-SOX2OT at different doses for 4 weeks by tail vein injection, followed by left cardiac ventricle injection of A549 cells (results of cell viability test are presented in Figure S2). Histological analysis showed that lncRNA-SOX2OT-enriched exosomes promoted BoM in NSCLC (Fig. 2E).

### LncRNA-SOX2OT-enriched exosomes regulate the expression of RANKL, OPG, TGF-β1 and pTHrP

**Fig. 3:**
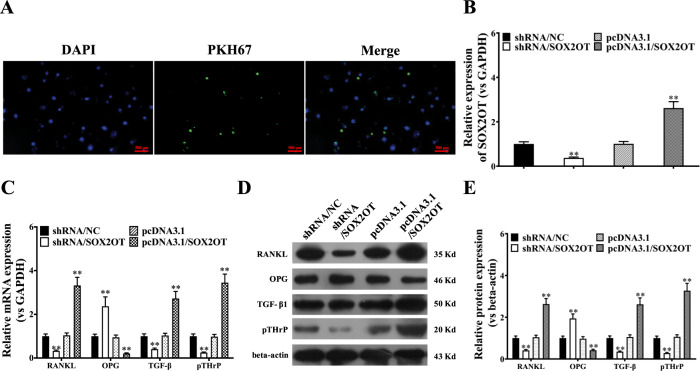
LncRNA-SOX2OT-enriched exosomes regulate the expression of RANKL, OPG, TGF-β1 and pTHrP. **A** To confirm that the exosomes could be taken up by recipient cells, the A549 cell-derived exosomes were stained with the red fluorescent dye PKH-26 at 37 °C for 1 h, washed with phosphate-buffered saline (PBS), followed by centrifugation at 110,000 × *g* and 4 °C for 70 min to remove the residual PKH-26 dye. RAW264.7 cells were co-incubated with PKH67-labelled exosomes (green) derived from A549 cells. Scale bar, 500 μm. **B** Real-time PCR assay detected the level of lncRNA-SOX2OT in RAW264.7 cells pre-treated with exosomes derived from A549 cells with different lncRNA-SOX2OT expression levels. GAPDH was used as the internal control. **C** Real-time PCR assay measured the mRNA expression of TGF-β, pTHrP, RANKL and OPG in RAW264.7 cells treated with exosomes from lncRNA-SOX2OT-knockdown or lncRNA-SOX2OT-overexpressing A549 cells. GAPDH was used as the internal control. **D**, **E** Western blot assays were performed to measure the protein expression of TGF-β, pTHrP, RANKL and OPG in RAW264.7 cells treated with exosomes from lncRNA-SOX2OT-knockdown or lncRNA-SOX2OT-overexpressing A549 cells. Beta-actin was used as a referred protein. *, *P* < 0.05; **, *P* < 0.01 (t test).

To confirm that the exosomes could be taken up by the recipient cells, RAW264.7 cells were co-incubated with PKH67-labelled exosomes (green) derived from A549 cells. As shown in Fig. 3A, green fluorescence was observed in the cytoplasm and around the nuclei of RAW264.7 cells after exposure to A549-derived exosomes, demonstrating effective uptake of PKH67-labelled exosomes by RAW264.7 cells. Next, we examined the level of lncRNA-SOX2OT in RAW264.7 cells pre-treated with exosomes derived from A549 cells with different lncRNA-SOX2OT expression levels. Real-time PCR results demonstrated that the transcript level of lncRNA-SOX2OT was low in the cytoplasm of RAW264.7 cells treated with exosomes from lncRNA-SOX2OT-knockdown A549 cells but significantly higher in the cytoplasm of RAW264.7 cells pre-treated with exosomes from lncRNA-SOX2OT-overexpressing A549 cells (Fig. 3B). Subsequently, we investigated whether lncRNA-SOX2OT-enriched exosomes affect osteoclast differentiation by regulating TGF-β/pTHrP/RANKL signalling. The real-time PCR assay showed reduced mRNA expression of TGF-β, pTHrP and RANKL but increased mRNA expression of OPG in RAW264.7 cells pre-treated with exosomes from lncRNA-SOX2OT-knockdown A549 cells, but the opposite results were seen in RAW264.7 cells pre-treated with exosomes from lncRNA-SOX2OT-overexpressing A549 cells (Fig. 3C). Western blot data were consistent with the real-time PCR results (Fig. 3D, E). Thus, lncRNA-SOX2OT-enriched exosomes regulated the TGF-β/pTHrP/RANKL signalling pathway in osteoclasts.

### LncRNA-SOX2OT Functions as a ceRNA by Targeting miR-194-5p in Osteoclasts

**Fig. 4:**
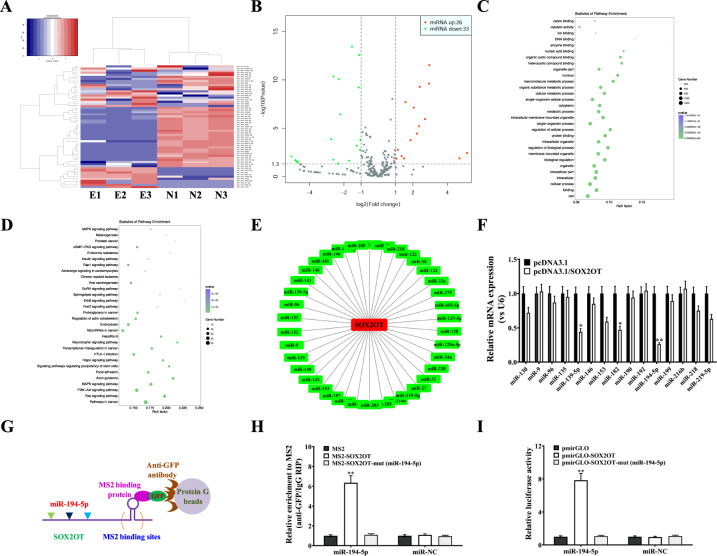
LncRNA-SOX2OT functions as a ceRNA regulating miR-194-5p in osteoclasts. **A** Cluster analyses of the differentially expressed miRNAs in the plasma of three NSCLC patients without metastasis (N1, N2, and N3) and three NSCLC patients with BoM (E1, E2, and E3). Red represents high gene expression, and blue represents low gene expression. The colour brightness of each band reflects the difference in multiples (log 2(AR/N)). **B** Volcano graph of miRNA differential expression analysis among samples (N1, N2, and N3; E1, E2, and E3). The abscissa represents the miRNA expression fold change in different samples, and the ordinate represents statistical significance of the miRNA expression difference. The red dots indicate the significant upregulation of miRNA expression, and the green dots indicate the significant downregulation of miRNA expression. **C**, **D** Kyoto Encyclopaedia of Genes and Genomes (KEGG) analysis was performed to identify the differentially expressed miRNAs involved in biological pathways. **E** An interaction network diagram was constructed between lncRNA-SOX2OT and the differentially expressed miRNAs using online programs including microRNA.org, star-Base v2.0 and MirTarget2. **F** Real-time PCR assay validated the RNA sequencing and target prediction results. U6 was used as the internal control. **G**, **H** MS2-RIP assay and real-time PCR assay detected the endogenous association of miR-194-5p with lncRNA-SOX2OT. U6 was used as the internal control. **I** Luciferase activities were analysed in RAW264.7 cells co-transfected with miR-194-5p and luciferase reporters containing the empty vector, lncRNA-SOX2OT wild-type transcript or lncRNA-SOX2OT mutant transcript. The final data are presented as the relative ratio (firefly luciferase activities/Renilla luciferase activities). Data are representative of three biological replicates, and each experiment was performed at least three times independently. *, *P* < 0.05; **, *P* < 0.01 (t test).

Several lncRNAs have been discovered to function as ceRNAs (competing endogenous RNAs) by binding to target miRNAs and regulating downstream gene expression. To identify potential miRNA targets of lncRNA-SOX2OT, we performed RNA sequencing and analysed differentially expressed miRNAs (Fig. 4A). Twenty-six miRNAs were upregulated, while 33 miRNAs were downregulated (Fig. 4B). By GO function and KEGG pathway enrichment analyses (Fig. 4C, D), numerous miRNAs were identified to be associated with osteoclast differentiation. In addition, by bioinformatic analysis, an important interaction network between lncRNA-SOX2OT and potential miRNAs was constructed (Fig. 4E). Further real-time PCR assays validated all the potential miRNA targets of lncRNA-SOX2OT, and miR-194-5p was chosen for further investigation since it had the largest fold change (Fig. 4F).

To confirm the interaction between lncRNA-SOX2OT and miR-194-5p, RIP was performed to pull down miRNAs binding to endogenous lncRNA-SOX2OT (Fig. 4G). Real-time PCR demonstrated that the lncRNA-SOX2OT immunoprecipitants derived from RAW264.7 cells showed significant enrichment of miR-194-5p compared to those for the control vector (MS2), IgG, miR-67 (a non-targeting miRNA), and lncRNA-SOX2OT with mutations in the miR-194-5p target sites (Fig. 4H). Additionally, to confirm the specific connection between lncRNA-SOX2OT and miR-194-5p, dual-luciferase reporters comprising the 5′ end of lncRNA-SOX2OT, which included either the wild-type (WT) or the mutated miR-194-5p binding sites, were constructed. Subsequently, when miR-194-5p was co-transfected with pmirGLO-SOX2OT, the luciferase activity of pmirGLO-SOX2OT was decreased, but the luciferase activities of the control vector pmirGLO and pmirGLO-SOX2OT-mut (miR-194-5p) had little effect (Fig. 4I). Taken together, the above results suggest that miR-194-5p is a direct target of lncRNA-SOX2OT in osteoclasts.

### miR-194-5p inhibits RAC1 expression

**Fig. 5:**
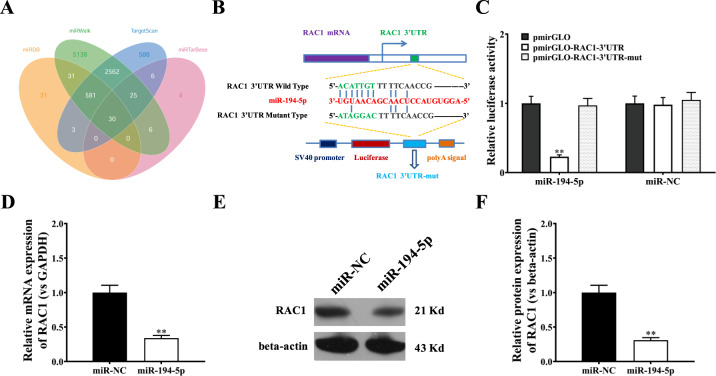
miR-194-5p inhibits RAC1 expression. **A** Interaction network diagram between the miR-194-5p family and differentially expressed mRNAs using microRNA.org, star-Base v2.0, miRDB and TargetScan. **B** The predicted binding sites of miR-194-5p in the RAC1 3′UTR. The red nucleotides are the seed sequences of miRNAs. The sequence of the RAC1 3’UTR with wild-type (WT) versus mutant (mut) miR-194-5p target sites is shown. **C** Luciferase reporter activities of wild-type RAC1 3′UTR, mutant RAC1 3′UTR, and empty construct reporters in RAW264.7 cells overexpressing miR-194-5p. **D** Real-time PCR assay evaluated RAC1 mRNA expression in RAW264.7 cells transfected with miR-194-5p or miR-NC. GAPDH was used as the internal control. **E** Western blot analysis of RAC1 protein expression in RAW264.7 cells transfected with miR-194-5p or miR-NC. Beta-actin was used as a referred protein. Data are representative of three biological replicates, and each experiment was performed at least three times independently. *, *P* < 0.05; **, *P* < 0.01 (t test).

The aforementioned observations prompted us to identify potential targets of miR-194-5p. The bioinformatic analysis revealed many relevant targets of miR-194-5p (Fig. 5A) including RAC1. Figure 5B depicts obvious binding sites of miR-194-5p in the 3′UTR of the RAC1 mRNA transcript. Therefore, we focused on miR-194-5p to investigate its role in regulating the potential target gene RAC1. We first generated luciferase reporter constructs containing the RAC1 3′UTR with or without a miR-194-5p binding site-null mutant sequence (Fig. 5B) and then tested their luciferase activity in RAW264.7 cells by co-transfection with a miR-194-5p-expressing vector. The results demonstrated a clear inhibitory effect of miR-194-5p on RAC1 3′UTR reporter activity (Fig. 5C). Additional analysis confirmed that miR-194-5p negatively impacted the transcriptional and translational levels of RAC1 (Fig. 5C–E).

### Effects of exosomal lncRNA-SOX2OT on osteoclast-related proteins and pathways in an NSCLC BoM model

**Fig. 6:**
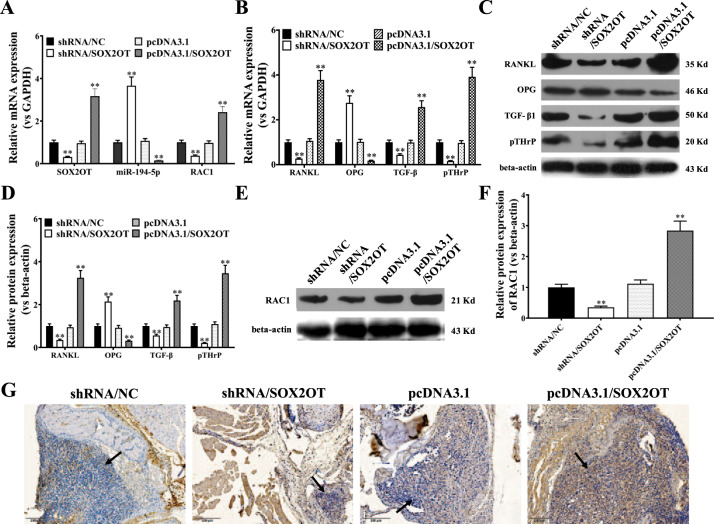
Effects of exosomal lncRNA-SOX2OT on osteoclast-related proteins and pathways in a lung cancer BoM model. **A** Real-time PCR assays for lncRNA-SOX2OT, miR-194-5p, and RAC1 in bone metastatic lesions treated with lncRNA-SOX2OT-knockdown exosomes or lncRNA-SOX2OT-enriched exosomes compared with their respective controls. GAPDH or U6 was used as the internal control. **B** Real-time PCR analysis for the mRNA levels of TGF-β, pTHrP, RANKL, and OPG in bone metastatic lesions treated with lncRNA-SOX2OT-knockdown exosomes or lncRNA-SOX2OT-enriched exosomes compared with that in their respective controls. GAPDH was used as the internal control. **C**, **D** Western blot analysis for the protein expression levels of TGF-β, pTHrP, RANKL, and OPG in bone metastatic lesions treated with lncRNA-SOX2OT-knockdown exosomes or lncRNA-SOX2OT-enriched exosomes compared with their respective controls. Beta-actin was used as a referred protein. **E**, **F** Western blot analysis for the RAC1 protein in bone metastatic lesions treated with lncRNA-SOX2OT-knockdown or lncRNA-SOX2OT-enriched exosomes compared with that in their respective controls. Beta-actin was used as a referred protein. **G** Immunohistochemical analysis for the expression of the RAC1 protein in bone metastatic lesions treated with lncRNA-SOX2OT-knockdown or lncRNA-SOX2OT-enriched exosomes compared with that in their respective controls. Black arrows indicate the metastatic foci and cancerous cells in the representative images. Scale bar, 200 μm. Data are representative of three biological replicates, and each experiment was performed at least three times independently. *, *P* < 0.05; **, *P* < 0.01 (t test).

Since lncRNA-SOX2OT was found to regulate miR-194-5p, RAC1 and TGF-β/pTHrP/RANKL signalling in vitro, we further examined the effects of exosomal lncRNA-SOX2OT on osteoclast-related proteins and pathways in the NSCLC BoM model. Real-time PCR demonstrated that, compared to corresponding controls, the expression of lncRNA-SOX2OT and RAC1 was lower in bone metastatic lesions of mice treated with lncRNA-SOX2OT-knockdown exosomes and higher in those treated with lncRNA-SOX2OT-enriched exosomes (Fig. 6A). Furthermore, compared to corresponding controls, miR-194-5p expression was higher in bone metastatic lesion tissues treated with lncRNA-SOX2OT-knockdown exosomes and lower in those treated with lncRNA-SOX2OT-enriched exosomes (Fig. 6A). In addition, compared to corresponding controls, we found that the expression of TGF-β, pTHrP and RANKL was lower in bone metastatic lesion tissues treated with lncRNA-SOX2OT-knockdown exosomes and higher in those treated with lncRNA-SOX2OT-enriched exosomes (Fig. 6B). Likewise, compared to corresponding controls, the expression of OPG was higher in bone metastatic lesion tissues treated with lncRNA-SOX2OT-knockdown exosomes and lower in those treated with lncRNA-SOX2OT-enriched exosomes (Fig. 6B). Western blot data were consistent with the real-time PCR results shown in Fig. 6B (Fig. 6C, D). Moreover, compared to corresponding controls, the RAC1 protein level was lower in bone metastatic lesion tissues treated with lncRNA-SOX2OT-knockdown exosomes but higher in those treated with lncRNA-SOX2OT-enriched exosomes (Fig. 6E, F). IHC data further confirmed the Western blot results shown in Fig. 6E (Fig. 6G).

## Discussion

Bone metastasis (BoM) is one of the most significant challenges facing oncologists and NSCLC patients since it can cause severe symptoms, life-threatening complications and shorten overall survival [1–4]. Hence, it is of great importance to study the molecular mechanisms and potential therapeutic targets of BoM in NSCLC.

In NSCLC, a great majority of the BoM cases that occur are osteolytic metastasis cases, and the most frequently reported underlying mechanism of osteolytic metastasis is RANK/RANKL signalling pathway-mediated osteoclast maturation [24]. In contrast, OPG can act as a receptor by binding to RANKL, thereby preventing its action on osteoclasts and their precursors [25]. Moreover, TGF-β/pTHrP-mediated overexpression of RANKL can enhance osteolytic BoM in NSCLC [9, 10]. To understand the upstream regulatory mechanism of the TGF-β/pTHrP/RANKL signalling pathway, we focused on exosomes, which can mediate communication between cells and affect the biological function of receptor cells [26]. Exosomes are microvesicular bodies secreted by living cells and approximately 30-100 nm in diameter. Exosomes contain a large number of functional proteins, DNA, mRNA, and noncoding RNAs (miRNA, lncRNA, piRNA, circRNA, etc.) and can be transported between cells to regulate the biological functions of cells [12–14]. Tumour-derived exosomes perform important biological functions in facilitating tumour growth and metastasis in most tumour types [27]. Tumour-derived exosomes function as extracellular organelles that deliver information during cell-cell communication and remodel the tumour microenvironment [28–31]. In particular, exosomes can transport various DNAs, RNAs and proteins between tumour cells and neighbouring cells to affect tumour microenvironments and the growth, invasion and metastasis of tumour cells [32].

To date, there are few published studies on exosomes in the process of BoM, and the effect of miRNAs in exosomes on BoM has only recently been reported [15, 33]. Reports on the effects of exosomal lncRNAs on bone metastases are relatively rare. LncRNA-SOX2OT is a recently characterised tumour-promoting lncRNA that plays critical roles in multiple types of cancers [16–21]. In particular, lncRNA-SOX2OT expression was found to be upregulated in tissues and serum samples from NSCLC patients, and it was closely related to the prognosis of NSCLC patients [22]. Furthermore, lncRNA-SOX2OT was also found to be enriched in exosomes from some cancers including NSCLC [23]. In addition, some studies have partially clarified the molecular mechanism involving exosomal lncRNA-SOX2OT in tumorigenesis [16]. In our study, the expression of exosomal lncRNA-SOX2OT was abnormally increased in the peripheral blood of NSCLC patients with BoM, and the levels of lncRNA-SOX2OT in NSCLC patient-derived exosomes were inversely correlated with the overall survival of NSCLC patients, indicating that exosomal lncRNA-SOX2OT could potentially serve as a powerful prognostic biomarker. Moreover, lncRNA-SOX2OT was detected in the exosomes of multiple NSCLC cell lines, including A549, H23, H358, H2030, H1299 and H1155 (Figure S3), providing evidence that lncRNA-SOX2OT might play significant roles in the progression of NSCLC.

Subsequently, we investigated the function and mechanism of exosomal lncRNA-SOX2OT in NSCLC progression, especially how exosomal lncRNA-SOX2OT affected osteolytic BoM in NSCLC. The result from the in vivo tracing experiments showed that NSCLC-derived exosomes were distributed in the lungs, liver, brain and bone marrow, and subsequent experimental BoM models demonstrated that lncRNA-SOX2OT-enriched exosomes promoted BoM in NSCLC. Further in vitro studies demonstrated that knockdown of lncRNA-SOX2OT expression in NSCLC cell lines decreased the cellular and exosomal expression of lncRNA-SOX2OT and reduced the delivery of exosomal lncRNA-SOX2OT to recipient RAW264.7 cells, which demonstrated important communication between tumour cells and osteoblasts via the transport of exosomal lncRNA-SOX2OT. Since RANK/RANKL signalling and the TGF-β/pTHrP-mediated overexpression of RANKL have been reported to be closely associated with osteolytic BoM in NSCLC [6, 9, 10, 24, 25], we performed additional experiments to probe the effect of exosomal lncRNA-SOX2OT on the above-mentioned signalling pathways. The results demonstrated that lncRNA-SOX2OT-enriched exosomes could regulate the expression of RANKL, OPG, TGF-β1 and pTHrP, which further confirmed the effects of exosomal lncRNA-SOX2OT on osteolytic BoM in NSCLC. The naturally secreted decoy receptor OPG is known to induce osteoclast apoptosis and inhibit bone resorption [7, 8]. In addition to OPG, other tumour-secreted proteins, such as osteopontin [34] and CXCL14 [35], have critical roles in the regulation of bone metastasis in lung cancer, either through exosomal trafficking or directly through paracrine signalling. Bone metastasis is a complicated process that involves multiple interconnected and independent signalling pathways and regulatory molecules, and the current study identified only one of them.

As the next step, we further investigated how exosomal lncRNA-SOX2OT enhanced the activation of osteoclasts. Multiple lncRNAs have been discovered to function as ceRNAs to regulate target miRNA aggregation and biological functions, thereby affecting tumorigenesis and disease progression [36]. In this study, we used the miRcode online website to identify potential miRNA targets of lncRNA-SOX2OT and predicted that exosomal lncRNA-SOX2OT might recognise miR-194-5p. By adopting a luciferase reporter construct with a mutation in the miR-194-5p recognition sequence for lncRNA-SOX2OT, we verified the direct relationship between lncRNA-SOX2OT and the target miR-194-5p. Accordingly, we conclude that, in NSCLC cells, exosomal lncRNA-SOX2OT is an upstream regulatory gene of miR-194-5p.

Finally, we attempted to identify major targets of miR-194-5p. Through bioinformatic predictions and experiments in animal models, we discovered that as a regulatory miRNA, miR-194-5p targeted the downstream gene RAC1 to repress RAC1 expression. RAC1 (ras-related C3 botulinum toxin substrate 1) is a member of the Rho GTPases, which transmit signals originating from the integrin signalling pathway and the Wnt signalling pathway [37, 38]. Previous reports indicated that deletion of RAC1 suppressed osteoblast differentiation [39, 40]. Our studies demonstrated that NSCLC cell-derived exosomal lncRNA-SOX2OT could regulate RAC1 expression through miR-194-5p in osteoclasts. However, the relationship between miRNA-194-5p-mediated RAC1 expression regulation and the TGF-β/pTHrP/RANKL signalling pathway is not clear at present and requires further investigation. Moreover, due to the significance of RAC1 in tumour metastasis, therapeutic inhibition of RAC1 may be of significant clinical importance in cancer treatment. There are several RAC1 inhibitors, such as ketorolac [41], EHop-016 [42] and NSC23766 [43], which are currently under development.

Additionally, we also recognise the limitations of our current study. Tumour-stroma interactions are critically involved in the progression of lung cancer metastasis. For example, heterotypic tumour cell-CAF interactions play significant roles in lung adenocarcinoma metastasis [44]; VCAM-1 secreted from CAFs enhances the growth and invasion of lung cancer cells by activating AKT and MAPK signalling [45]; cancer-associated fibroblasts activated by miR-196a promote the migration and invasion of lung cancer cells [46]; microsomal prostaglandin E synthase-1 promotes lung metastasis via SDF-1/CXCR4-mediated recruitment of CD11b^+^ Gr1^+^ MDSCs from bone marrow [47]; lung cancer-derived succinate promotes macrophage polarisation and cancer metastasis via succinate receptors [48], etc.

**Fig. 7:**
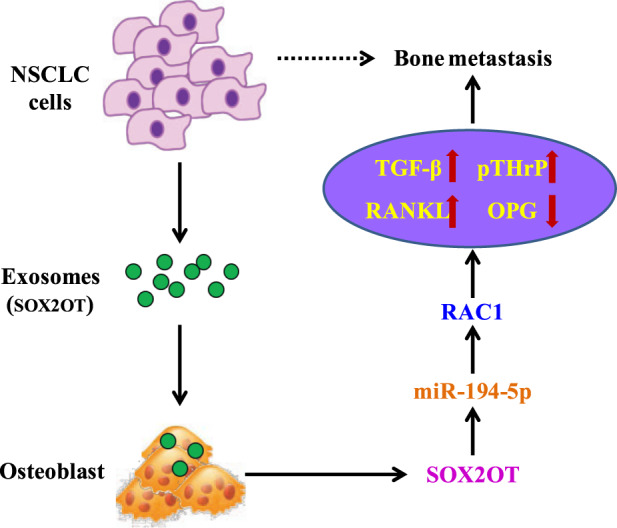
Schematic diagram of this study. A schematic model showing that tumour-derived exosomal lncRNA-SOX2OT promotes the bone metastasis of the lung cancer by targeting the RAC1 signalling pathway through miRNA-194-5p in osteoclasts.

In summary, our findings demonstrate that NSCLC cell-derived exosomal lncRNA-SOX2OT regulates osteoclast differentiation and promotes BoM of NSCLC cells by targeting the miRNA-194-5p-mediated RAC1 signalling pathway and regulating the TGF-β/pTHrP/RANKL signalling pathway in osteoclasts (Fig. 7). Our study will enrich our understanding on the molecular basis of lung cancer BoM and provide a mechanistic basis for targeted therapy for lung cancer BoM.

## Materials and methods

### Human samples and cell lines

Plasma samples from a total of 142 advanced NSCLC patients (including 58 cases with BoM) were collected between January 2017 and December 2018 at Fudan University Shanghai Cancer Center. The baseline characteristics of 142 advanced NSCLC patients are presented in Table 1. Human A549 cell lines and RAW264.7 cells were obtained from American Type Culture Collection (ATCC) and cultured in RPMI 1640 medium and DMEM, respectively. HEK293T cells from ATCC were cultured in DMEM medium. All media were supplemented with 10% foetal bovine serum (FBS) and 1% penicillin-streptomycin. All cells were cultured at 37 °C in a 5% CO_2_ humid atmosphere. All procedures were approved by the Ethics Committee of the Fudan University Shanghai Cancer Center. Informed consent for publication was obtained from all subjects.

**Table 1:** Demographic and baseline clinical characteristics of the NSCLC patients.

Patient characteristics	Number of patients (*n* = 142)
Age (years)	
≤50	53
>50	89
Sex	
Male	83
Female	59
Smoking history	
Yes or ever	51
No	91
Number of primary lesions	
Single	33
Multiple	109
Pathological type	
Adenocarcinoma	93
Nonadenocarcinoma	49
Bone metastasis	
Yes	58
No	84
ECOG PS	
0–1	87
≥2	55

### Exosome isolation and characterisation

Plasma samples or cell culture medium were pre-treated by differential centrifugation at 300 × *g* for 10 min to remove the residual cells, 2,000 × *g* for 15 min to remove the cell debris, and 10,000 × *g* for 30 min to remove the large molecules. All the processes of centrifugation were performed at 4 °C. Next, the exosomes containing pellets were obtained by ultracentrifugation at a speed of 100,000 × *g* for 70 min (Beckman 70Ti rotor). The pellets were washed with PBS, followed by ultracentrifugation at 100,000 × *g* for another 70 min. Subsequently, the morphology of the collected exosomes was examined by transmission electron microscopy (TEM). An LM10 nanoparticle characterisation system (NanoSight, Malvern Instruments) was used to measure the number and size distribution of the collected exosomes.

### Exosome labelling and fluorescence microscopy

RAW264.7 cells (8 × 10^4^ cells/ml) were seeded in 12-well plates. The red fluorescent dye PKH-26 (Invitrogen) was used to label the A549 cell-derived exosomes at 37 °C for 1 h, and the labelled exosomes were subsequently washed with phosphate-buffered saline (PBS), followed by centrifugation at 110,000 × *g* and 4 °C for 70 min to remove the residual PKH-26 dye. RAW264.7 cells were incubated with PKH-26-labelled exosomes for 4 h, followed by fixation with 4% paraformaldehyde for 1 h. 4’,6-diamidino-2-phenylindole (DAPI) was used for nuclear staining. The incorporation of PKH-26-labelled exosomes into the targeted RAW264.7 cells was analysed by fluorescence microscopy (Zeiss AG, Germany).

### Plasmid construction and cell transfection

The human SOX2OT gene or the mutant SOX2OT gene (mutations in the targeting site for miR-194-5p) was chemically synthesised and subcloned into the blank vector pcDNA3.1-MS2. Resultant constructs were named pcDNA3.1-MS2-SOX2OT and pcDNA3.1-MS2-SOX2OT-mut (miR-194-5p), respectively. The 1,000-nt gene fragment from the 5’ end of SOX2OT or SOX2OT-mut (miR-194-5p) was amplified by routine PCR and subcloned into the pmirGLO vector (Promega). The resultant recombinant plasmids carrying wild-type or mutant SOX2OT were used for luciferase reporter analysis as described below.

MiR-194-5p mimics, control negative miRNA mimics, miR-194-5p inhibitors, and recombinant adenoviruses overexpressing EBLN3P or control adenoviruses were prepared by GeneChem (Shanghai, China). The recombinant gene silencing plasmid pLV4-shRNA/SOX2OT was constructed. All transient transfection experiments were performed using Lipofectamine 3000 reagent (Invitrogen).

### Real-time PCR

For RAC1 gene quantification and lncRNA-SOX2OT and miR-194-5p expression analysis, total RNA was extracted using a TRIzol kit (Invitrogen). A NanoDrop ND-1000 instrument (NanoDrop) was used to measure the concentrations of the RNAs. A SuperScript® III RT-PCR kit (Life Technologies) was used to produce cDNA. The primers for real-time PCR were prepared as follows: RAC1: 5′-CCCCATTCTTGTTCAGATT-3′ (sense), 5′-TGCTTTACGCATCTGAGAACT-3′ (antisense); lncRNA-SOX2OT: 5′-GTTCATGGCCTGGACTCTCC-3′ (sense), 5′-ATTGCTAGCCCTCACACCTC-3′ (antisense); miR-194-5p: 5′-CCATGATT CCTTCATATTTGC-3′ (sense), 5′-GCAAATATGAAGGAATCATGG-3′ (antisense); GAPDH: 5′-GGTGGTCTCCTCTGACTTCAACA-3′ (sense) and 5′-CCAAATTCGTTGTCATACCAGGAAATG-3′ (antisense); and U6: 5′-CTCGCTTCGGCAGCACA-3′ (sense), 5′-AACGCTTCACGAATTTGCGT-3′ (antisense). The parameters for PCR amplification were as follows: 2 min at 95 °C, followed by 40 cycles of 15 s at 95 °C and 30 s at 60 °C. The real-time quantitative PCR data were analysed using the threshold cycle (Ct), and the relative gene expression levels were calculated by the 2^-△△Ct^ method.

### RNA immunoprecipitation analysis

A549 cells were co-transfected with pMS2-GFP and pcDNA3.1-MS2 (or pcDNA3.1-MS2-SOX2OT, or pcDNA3.1-MS2-SOX2OT-mut (miR-194-5p)) for 48 h. Total RNA was extracted from transfected A549 cells to perform an RIP assay using an anti-GFP antibody (Roche) and the Magna RIP™ RNA-Binding Protein IP Kit (Millipore, USA).

### Luciferase reporter analysis

HEK293T cells (8,000/well) or A549 cells (12,000/well) were seeded in 96-well plates and co-transfected with 50 nmol/L miR-194-5p mimic (or NC), 50 ng of luciferase reporter plasmid, and 5 ng of pRLCMV Renilla luciferase reporter plasmid using Lipofectamine 3000 reagent for 48 h. Next, the dual-luciferase reporter assay kit (Promega) was used to measure the Renilla and firefly luciferase activities. The final data are presented as the relative ratio (firefly luciferase activity/Renilla luciferase activity).

### Western blotting

Protein was extracted from A549 cells, separated by 10% SDS/PAGE, and transferred to PVDF membranes (Millipore, USA). Next, the membranes were incubated with the respective primary antibodies at 4 °C overnight, followed by three washes in PBS. Then, the membranes were incubated with HRP-conjugated anti-rabbit or anti-mouse secondary antibodies at 37 °C for 1 h, and the protein bands were detected using an Odyssey scanning system. An anti-TGF-β rabbit mAb (3709, CST), anti-pTHrP rabbit mAb (orb303841, Biobyt), anti-RANKL rabbit mAb (3959, CST), anti-OPG mouse mAb (sc-390518, Santa Cruz), anti-RAC1 rabbit polyclonal antibody (24072-1-AP, Proteintech), and anti-beta-actin rabbit mAb (4970, CST) were purchased. For protein detection in the exosomes, the extracted exosomes were suspended in SDS lysis buffer, and the concentration of the exosomal proteins was determined by the BCA assay. The exosome-associated protein markers CD63 (ab193349, Abcam), CD9 (ab92726, Abcam) and HSP70 (4876, CST) were detected by the Western blot, as described above.

### Cell viability assay

A 5 × 10^4^ cell/ml A549 cell suspension was seeded in a 96-well plate. After appropriate treatments were administered for 48 h, diluted MTT was added to each well of A549 cells and incubated for 1 h. Next, DMSO at a 100% concentration was added to each well of A549 cells, and then the absorbance of each well was measured at 570 nm. All experiments were performed at least three times.

### Establishment of a BoM model of lung cancer

A total of 4- to 6-week-old BALB/c nude mice (weighing 20 g ± 2 g, and 1:1 male:female ratio) were randomly divided into four groups receiving shRNA/NC exosomes, shRNA/SOX2OT exosomes, pcDNA3.1 exosomes or pcDNA3.1/SOX2OT exosomes. For each group, 8 mice were included. Each BALB/c nude mouse received 10 μg of exosomes by tail vein injection every other day for 4 weeks. A549 cells were used to establish the lung cancer BoM model in BALB/c nude mice. A549 cells with appropriate modifications were digested and collected in a centrifuge tube, and a cell suspension with a concentration of 1 × 10^7^ cell/mL was made with PBS. Trypan blue staining was performed to confirm that the cell viability was ≥95%. A total of 75 BALB/c nude mice were enrolled in the study. The mice were anaesthetised and inoculated into the left cardiac ventricle with 1 × 10^5^ A549 cells in 100 μL PBS. After 28 days, samples were collected from bone metastatic lesions for follow-up experiments. All the data were evaluated and classified blindly by two investigators. The experimental procedures were approved by the Animal Care Commission of Fudan University.

### HE and immunohistochemistry staining

Paraffin-embedded tissue samples from bone metastatic lesions were used for haematoxylin-eosin (HE) staining and immunohistochemistry (IHC) staining. For HE, tissue sections were deparaffinised and hydrated in distilled water, and Mayer’s haematoxylin (Lillie’s Modification) was applied to completely cover tissue sections. After 5 min of incubation, the slides were rinsed in two changes of distilled water to remove excess stain, and Bluing Reagent was applied to completely cover the tissue sections. After incubating for 10–15 s, the slides were rinsed in two changes of distilled water. Subsequently, the slides were dipped in absolute alcohol and excess was blotted off. Eosin Y Solution was applied to cover tissue section to excess and the slides were incubated for 2–3 min. The slides were rinsed using absolute alcohol, dehydrated in three changes of absolute alcohol, cleared and mounted in synthetic resin. For IHC, paraffin sections were dewaxed by sequentially submerging in xylene I (8 min), xylene II (8 min), absolute ethanol and xylene (1:1; 5 min), absolute ethanol (3 min), 95% ethanol (3 min), 85% ethanol (3 min) and 75% ethanol (3 min) for aqueous penetration, and antigen retrieval was performed by boiling in sodium citrate antigen retrieval solution at 100 °C for 20 min. After allowing the sections to cool, a 3% H_2_O_2_ solution was added dropwise, followed by incubation for 10 min at room temperature. The sections were then blocked with 5% BSA for 30 min and incubated with a primary antibody (RAC1, 24072-1-AP, Proteintech) diluted 1:1000 at 4 °C overnight. The next day, the sections were washed 3 times with PBS, incubated with secondary antibody (HRP-conjugated goat anti-rabbit IgG, ab150077, Abcam) at room temperature for 2 h, and then developed with a DAB staining solution (ab64238, Abcam). A DMI3000B microscope was used to assess the HE and IHC staining results.

### Statistical analysis

All the data from at least three independent experiments are presented as the mean ± standard deviation. A normality test (D’Agostino-Pearson) was used to analyse the distribution of all datasets. SPSS 24.0 software was employed to analyse the data using the two-tailed Student’s *t* test or one-way ANOVA method. *P* < 0.05 was considered statistically significant.

### Study approval

All procedures involving human subjects were performed with the approval of the ethics committee of the Fudan University Shanghai Cancer Center. Written informed consent was obtained from all patients. All animal protocols were approved by the Animal Care Commission of Fudan University.

## Supplementary information

Supplementary figure legends

Figure S1

Figure S2

Figure S3
