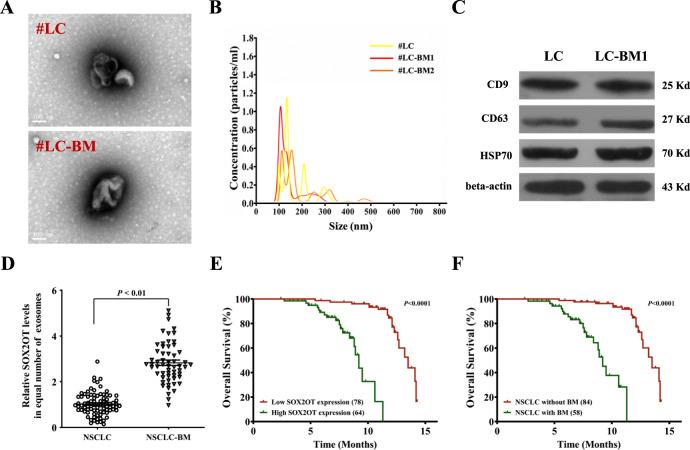# Correction: Tumour-derived exosomal lncRNA-SOX2OT promotes bone metastasis of non-small cell lung cancer by targeting the miRNA-194-5p/RAC1 signalling axis in osteoclasts

**DOI:** 10.1038/s41419-021-04399-9

**Published:** 2021-12-06

**Authors:** Jianjiao Ni, Xiaofei Zhang, Juan Li, Zhiqin Zheng, Junhua Zhang, Weixin Zhao, Liang Liu

**Affiliations:** 1 Department of Radiation Oncology, Fudan University Shanghai Cancer Center, Shanghai, China; 2 Department of Oncology, Shanghai Medical College, Fudan University, Shanghai, China; 3 Department of Radiation Oncology, Fudan University Shanghai Cancer Center Minhang Branch Hospital, Shanghai, China

**Keywords:** Non-small-cell lung cancer, Oncogenesis

Correction to: *Cell Death and Disease* 10.1038/s41419-021-03928-w, published online 2 July 2021

The original version of this article unfortunately contained a mistake in Fig. 1c. The correct figure can be found below. The authors apologize for the mistake. The original article has been corrected.

**Figure 1:**