# Supplementary material for: Tumour-derived exosomal lncRNA-SOX2OT promotes bone metastasis of non-small cell lung cancer by targeting the miRNA-194-5p/RAC1 signalling axis in osteoclasts
Source: Cell Death Dis. 2021 Jul 2;12(7):662. doi: 10.1038/s41419-021-03928-w (PMC8253828; doi:10.1038/s41419-021-03928-w)
Supplement: Supplementary file 1 — Supplementary figure legends [file 41419_2021_3928_MOESM1_ESM.docx]

**Supplementary information**

**Figure S1** Quantitative analysis results for Figure 1C. Western blot was performed to detect the expression of exosomal markers, including CD9, CD63 and HSP70. Beta-actin was used as a referred protein. Data are representative of three biological replicates.

**Figure S2** The cell viability assay for A549 was performed by Trypan blue staining before the building of bone metastasis model of lung cancer.

**Figure S3** Real-time PCR assay was performed to observe the lncRNA-SOX2OT level in the exosomes derived from human non-small cell lung cancer cell lines, including A549, H23, H358, H2030, H1299 and H1155. GAPDH was used as the internal control.
