# Supplementary figures and images for: Tumour-derived exosomal lncRNA-SOX2OT promotes bone metastasis of non-small cell lung cancer by targeting the miRNA-194-5p/RAC1 signalling axis in osteoclasts
Source: Cell Death Dis. 2021 Jul 2;12(7):662. doi: 10.1038/s41419-021-03928-w (PMC8253828; doi:10.1038/s41419-021-03928-w)

**Figure S1**

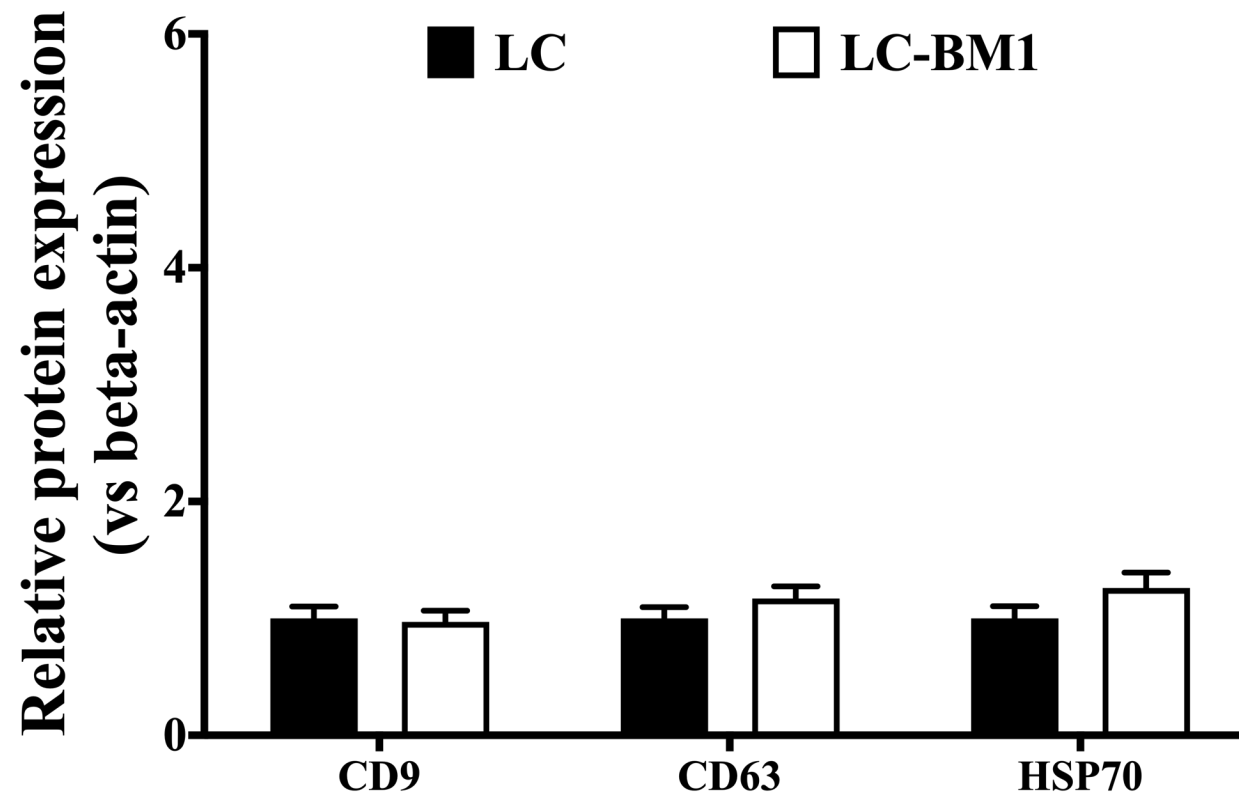

Supplement: Supplementary file 2 — Figure S1 [file 41419_2021_3928_MOESM2_ESM.pdf]

**Figure S2**

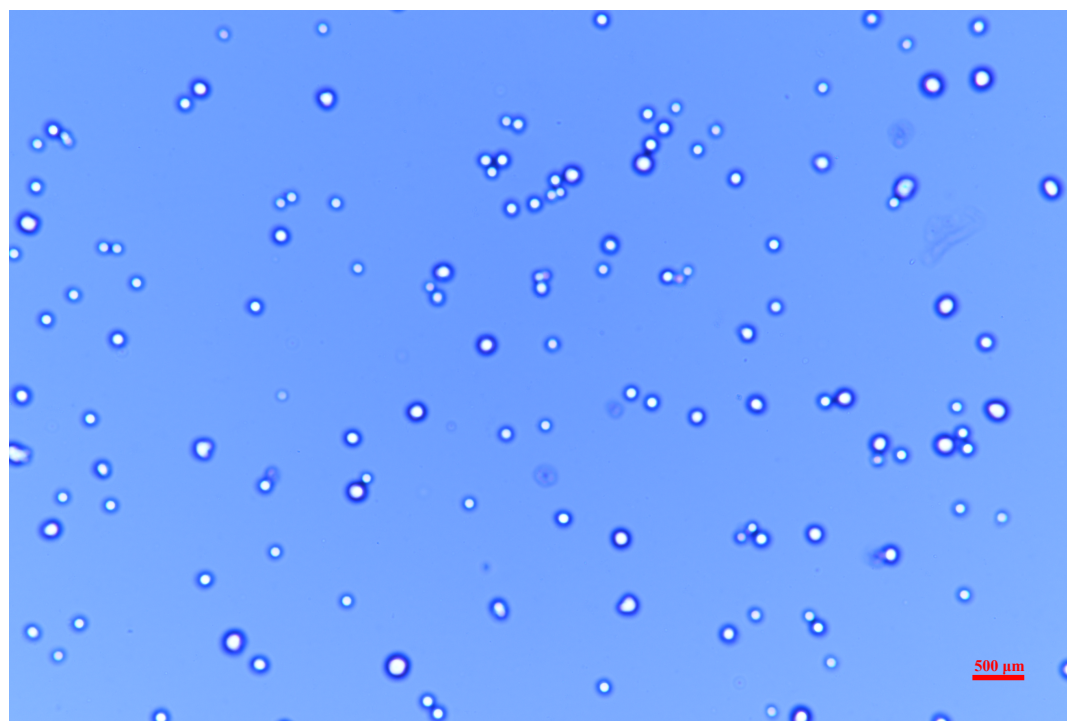

Supplement: Supplementary file 3 — Figure S2 [file 41419_2021_3928_MOESM3_ESM.pdf]

**Figure S3**

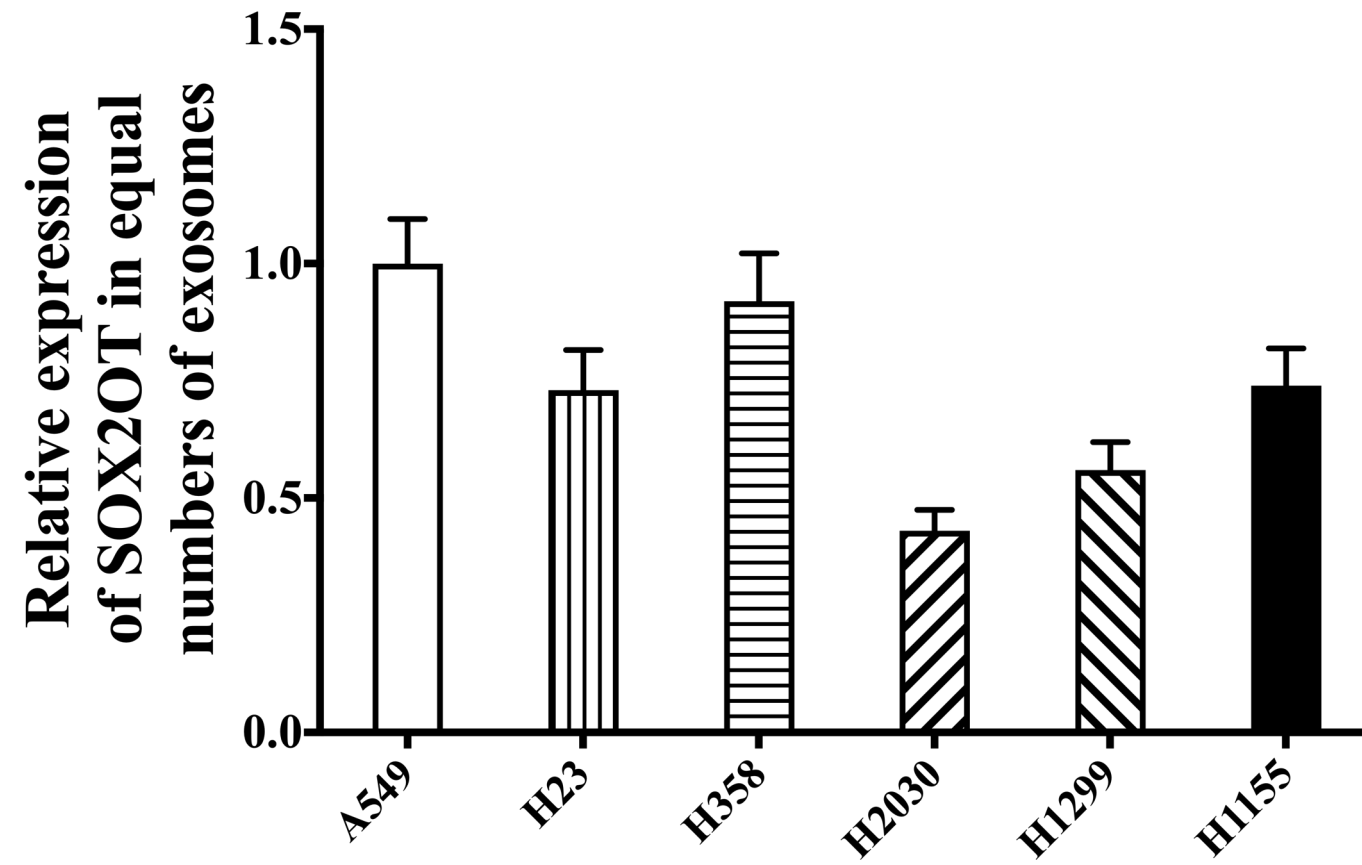

Supplement: Supplementary file 4 — Figure S3 [file 41419_2021_3928_MOESM4_ESM.pdf]
